# A quantitative fluorescence‐based steady‐state assay of DNA polymerase

**DOI:** 10.1111/febs.12760

**Published:** 2014-03-10

**Authors:** Max D. Driscoll, Julius Rentergent, Sam Hay

**Affiliations:** ^1^ Manchester Institute of Biotechnology and Faculty of Life SciencesUniversity of ManchesterUK

**Keywords:** DNA quantification, enzyme kinetics, fluorescence, Michaelis–Menten, PicoGreen, polymerase, steady‐state

## Abstract

Fluorescent dyes that bind DNA have been demonstrated as a useful alternative to radionucleotides for the quantification of DNA and the *in vitro* measurement of the activity of DNA polymerases and nucleases. However, this approach is generally used in a semi‐quantitative way to determine relative rates of reaction. In this report, we demonstrate a method for the simultaneous quantification of DNA in both its single‐strand and double‐strand forms using the dye PicoGreen. This approach is used in a steady‐state assay of DNA polymerase Klenow fragment exo^−^, where we determine *k*_cat_ and *K*_m_ values for the DNA polymerase that are in excellent agreement with literature values.

**Abbreviations**
KF: Klenow fragment of DNA polymerase I
PG: PicoGreen

## Introduction

The importance of DNA polymerase enzymes in both biology and biotechnology cannot be understated. Central to DNA replication and repair in all living organisms, polymerases have been studied for decades 1. Research into DNA polymerase kinetics dates back to the 1970s and 1980s and has typically used primer‐extension assays 2 or stopped or quench‐flow pre‐steady‐state methods 7 (for a review see 12). This has led to the employment of this group of enzymes in everyday laboratory protocols and techniques such as PCR and its derivatives and DNA sequencing. However, much of the kinetic characterization of DNA polymerase enzymes has historically relied on the use of radiological methods, which are now being phased out due to the inherent risks associated with the use of radioisotopes. Alternative methods are being adopted, frequently employing fluorescence detection 13, but these are often, at best, semi‐quantitative. For example, while it is possible, during a real‐time PCR reaction, to observe when a desired reaction has reached completion, and to make comparisons between different reactions, extracting reliable kinetic parameters (i.e. rate constants) is generally not possible. The utilization of fluorescent dyes to quantify DNA and to assay polymerase activity has been well established since the 1960s 19. Fluorescent nucleotide analogues have also been used to study DNA structure and polymerase mechanisms 22. However, to the best of our knowledge, these methods have not been employed in a simple assay for the elucidation of steady‐state kinetic parameters (i.e. *k*_cat_ and *K*_m_) which facilitates comparison with other kinetic studies performed using alternative approaches.

PicoGreen (PG) is a commercially available fluorescent dye that is often used for the quantification of dsDNA 24. It is similar in properties and structure to SYBR green 29, which has been used for many years to quantify DNA in such techniques as real‐time PCR and quantitative PCR 30. Fluorescent dyes have been shown to bind to DNA in a variety of different ways 34. PG has been shown both to intercalate into the double helix of DNA with a frequency of approximately one dye molecule per four base pairs of dsDNA and also to bind along the phosphate backbone surface using electrostatic interactions along with groove binding 35. PG displays high affinity to DNA, which lends itself to a large linear dynamic range. The fluorescence enhancement of PG upon association with dsDNA has been reported to be ~ 2000‐fold and detection of DNA concentrations ranging from low picomolar to micromolar by PG has been demonstrated as the *K*_d_ for PG/DNA is in the nanomolar range (dependent upon buffer conditions) 34. The fluorescence enhancement of PG when bound to ssDNA is significantly less than that when bound to dsDNA and the fluorescence sensitivity to dsDNA is not greatly affected by the presence of ssDNA 36. As a result, PG lends itself to studying the production of dsDNA from a single‐stand template, and therefore also to the study of DNA polymerase activity. Indeed, a PG‐based DNA polymerase assay has been demonstrated, but this approach has not yet progressed beyond the qualitative or comparative analysis of dsDNA production 14. Similarly, PG has also been employed to semi‐quantitatively monitor the hydrolysis of dsDNA catalysed by DNA nuclease 37.

In this report, we present a fluorescence‐based assay for the study of DNA polymerase kinetics based on the selective detection of dsDNA by PG. We focus on the steady‐state turnover of Klenow fragment (KF) exo^−^, which is a well‐characterized model polymerase 2. We have designed the assay to simplify the study of polymerases, with the removal of nucleotide analogues and the need to visualize results, indirectly, using gel electrophoresis. This assay can be used to obtain steady‐state parameters such as the apparent Michaelis–Menten constant (*K*_m_) and, as it is quantitative, the velocities *V*_max_ and *k*_cat_. The assay consumes relatively small quantities of DNA, nucleotides and enzyme and can be performed using a conventional fluorometer, simply first requiring the creation of a standard curve.

## Results and discussion

In order to monitor dsDNA production during a polymerase assay using a fluorophore such as PG, the fluorescence from PG bound to dsDNA (*f*_ds_) must be deconvoluted from the total observed fluorescence from the sample, *f*_obs_:

(1) $$f_{\mathrm{obs}}=f_{\mathrm{PG}}\left[\mathrm{PG}\right]+f_{\mathrm{dNTP}}\left[\mathrm{PG}\cdot \mathrm{dNTPs}\right]+f_{\mathrm{ds}}\left[\mathrm{PG}\cdot \mathrm{dsDNA}\right]+f_{\mathrm{ss}}\left[\mathrm{PG}\cdot \mathrm{ssDNA}\right]$$

**Figure 1:**
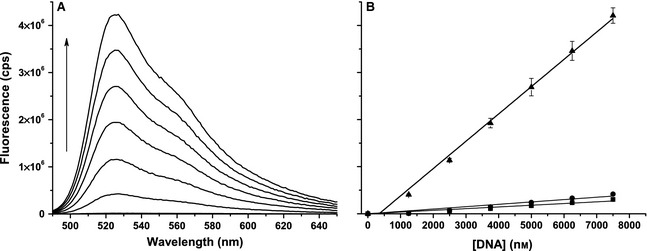
dsDNA and ssDNA quantification using PG. (A) Selected fluorescence emission spectra (excitation at 480 nm) as dsDNA (1 : 1 T7pR30 : T7pR30rc) is titrated into 3 μm PG. The arrow highlights the increase in emission at 525 nm with increasing [dsDNA]. (B) The dependence of the peak fluorescence emission at 525 nm on [dsDNA] (triangles) and [ssDNA] (T7pR30, circles; T7pR30rc, squares). Note that the DNA concentration is expressed in nM of nucleotides or base pairs (i.e. concentration of oligonucleotide × length). The data shown are averages of triplicate data sets and, using the standard deviations as errors, were fitted (excluding those at the origin) to a linear function to extract the *f*_ds_ and *f*_ss_ values given in the text. Note that while the data in (B) do not go through the origin – the linear dynamic ranges do not extend to [DNA] = 0 − this effect is not observed under assay conditions, as shown in Fig. 2.

Under the instrumental and buffer conditions used throughout this study (given in Materials and methods), we measured *f*_PG_ = 0.85 ± 0.23 counts per second (cps) per nM PG. By titrating dNTP into a solution of 3 μm PG (the PG concentration typically used throughout this study), a value of *f*_dNTP_ = 0.050 ± 0.002 cps·(nm dNTP)^−1^ was determined (data not shown). Next, 50‐mers of dsDNA and ssDNA were titrated into 3 μm PG to determine *f*_ds_ = 580 ± 18 cps·(nm dsDNA)^−1^ and *f*_ss_ = 36 ± 2 and 50 ± 3 cps·(nm ssDNA)^−1^ (Fig. 1). The dsDNA was made by annealing a 1 : 1 ratio of T7pR30 : T7pR30rc (Table 1) and the different values of *f*_ss_ are for the T7pR30 and T7pR30rc oligonucleotides, respectively. The variability in *f*_ss_ suggests that the magnitude of the fluorescence enhancement upon DNA binding to PG may have some sequence dependence.

**Table 1:** ssDNA oligonucleotide templates used in this work. The complementary sequences for the T7 primer are in bold and the T7 sequence in the reverse complement 50‐mer (T7pR30rc) is shown underlined. The nomenclature for the oligonucleotides refers to the T7 promoter sequence and the length of the random polynucleotide region of 30–100 nucleotides.

Oligonucleotide	Length	Sequence
T7pR30	50	5′‐TAC GGA TCC ATG CTA GTC CAT TAG CAG GTG **CCC TAT AGT GAG TCG TAT TA**‐3′
T7pR50	70	5′‐GTG GAC AGT CTG GTA TGT AGT CAG GCT CCA GGA GTC GCC TAT GCC AAC CT**C CCT ATA GTG AGT CGT ATT A**‐3′
T7pR80	100	5′‐GTA GCT GAC TTC TTC ACC ACA TCT ACC AAA GTG GGC AGT CTG GTA TGC AGT CAG GCT CCA GGA GTC GCC TAT GCC AAC CT**C CCT ATA GTG AGT CGT ATT A**‐3′
T7pR100	120	5′‐GAC TCG AAT GTG ACT CAG TGA TAG CTG ACT TCT TCA CCA CAT CTA CCA AAG TGG GCA GTC TGG TAT GCA GTC AGG CTC CAG GAG TCG CCT ATG CCA ACC T**CC CTA TAG TGA GTC GTA TTA**‐3′
T7pR30rc	50	5′‐TAA TAC GAC TCA CTA TAG GGC ACC TGC TAA TGG ACT AGC ATG GAT CCG TA‐3′

**Figure 2:**
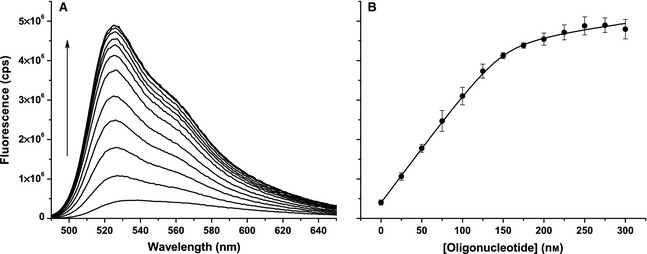
dsDNA and ssDNA discrimination using PG. (A) Fluorescence emission spectra as the ssDNA 50‐mer T7pR30 is titrated into 150 nm of its reverse complement (T7pR30rc) in the presence of excess (3 μm) PG. (B) The fluorescence at 525 nm versus [T7pR30]. The data shown are averages of triplicate data sets, using the standard deviations as errors, and are fitted to Eqn (2), with more detail given in the text.

The differential fluorescence enhancement of PG bound to dsDNA versus ssDNA (*f*_ds_/*f*_ss_) observed in Fig. 1B is 11–16, which is of the same order as previously reported 36. To alternatively determine *f*_ds_ and *f*_ss_ under conditions similar to those used during a polymerase assay, one ssDNA 50‐mer (T7pR50; Table 1) was titrated (with annealing) against its reverse complement (T7pR50rc). These data, shown in Fig. 2, were fitted to a quadratic function (Eqn (2)) to simultaneously determine *f*_ds_ and *f*_ss_.

(2) $$\begin{array}{cc} & f_{\mathrm{obs}}\approx f_{\mathrm{ds}}\left[\mathrm{dsDNA}\right]+f_{\mathrm{ss}}\left[\mathrm{ssDNA}\right]\\ & \left[\mathrm{dsDNA}\right]=\left\{length/2\right\}\left\{\left(\left[O_{1}\right]+\left[O_{2}\right]+K_{d}\right)-\sqrt{{\left(\left[O_{1}\right]+\left[O_{2}\right]+K_{d}\right)}^{2}-4\left[O_{1}\right]\left[O_{2}\right]}\right\}\\ & \left[\mathrm{ssDNA}\right]=length\left(\left[O_{1}\right]+\left[O_{2}\right]\right)-\left[\mathrm{dsDNA}\right] \end{array}$$

Here, *length* is the ssDNA (oligonucleotide) length (i.e. 50) and [O_1_] and [O_2_] are the concentrations of the T7pR50 and T7pR50rc oligonucleotides. *f*_dNTP_ and *f*_PG_ can also be omitted in the approximation of *f*_obs_ as their contributions are negligible. Fitting the data in Fig. 2B yielded *f*_ds_ = 562 ± 9 cps·nm^−1^, *f*_ss_ = 53 ±2 cps·nm^−1^ and *K*_d_ = 2.9 ± 0.7 nm. These values are comparable to those determined in Fig. 1 and yield *f*_ds_/*f*_ss_ = 10.6 ± 0.1. The observed *K*_d_ is in good agreement with previous reports 34. Consequently PG can be used to discriminate between dsDNA and ssDNA. The difference in fluorescence emission between PG bound to dsDNA and ssDNA (~ 11‐fold) is consistent between experiments. This is important to consider, as it has been shown that this difference in fluorescence enhancement is dependent upon buffer conditions 36.

The data in Figs 1 and 2 show that it is possible to accurately determine the concentration of dsDNA or ssDNA in reaction mixtures containing dsDNA, ssDNA and PG if the concentration of the other species is known. However, if we consider an idealized polymerase‐catalysed reaction (Eqn (3)), ssDNA and dNTPs are stoichiometrically converted to dsDNA.

(3) $${\mathrm{dsDNA}}_{n}\cdot {\mathrm{ssDNA}}_{m}+\mathrm{dNTP}\underset{\mathrm{polymerase}}{\;⟶}{\mathrm{dsDNA}}_{n+1}\cdot {\mathrm{ssDNA}}_{m-1}+\mathrm{PPi}$$

The change in *f*_obs_ as the reaction progresses (Δ*f*_obs_) then allows the determination of the amount of dsDNA produced (Δ[dsDNA]) without knowledge of the concentration of any of the species:

(4) $$\Delta f_{\mathrm{obs}}\approx \left(f_{\mathrm{ds}}-f_{ss}\right)\Delta \left[\mathrm{dsDNA}\right]=f_{\Delta }\Delta \left[\mathrm{dsDNA}\right]$$

We will use a value of *f*_Δ_ = 520 ± 30 cps·nm^−1^, which was determined using *f*_ds_ and *f*_ss_ values averaged between those determined in Figs 1 and 2. While this value will vary depending on the fluorometer and instrument parameters employed, it can be readily determined using the method described in Fig. 1 and/or Fig. 2. Note that if absolute values of *V*_max_ and *k*_cat_ are not required, then relative velocities can be readily determined by comparison with a reference reaction. This removes the necessity of the creation of a standard curve to determine *f*_Δ_ in Eqn (4).

**Figure 3:**
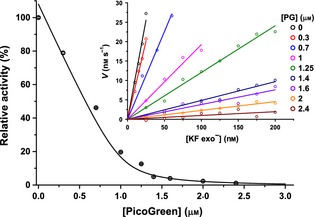
Steady‐state turnover in the presence of PG. The observed rate of steady‐state turnover of KF exo^−^ with 100 nm template DNA (1 : 2 T7pR30 : T7 primer) and 1 mm dNTPs in the presence of increasing PG at room temperature. Reactions containing 0, 0.3 and 0.7 μm PG were performed using the stopped method in Fig. 4 with the PG concentration made up to 3 μm immediately prior to measurement (to ensure sufficient PG to report on all the dsDNA produced). All other reactions were performed continuously. The inset shows the dependence of observed *V* on [KF exo^−^]. These data were each fitted to a linear function (solid lines) to determine the data shown in the main panel. The [PG] dependence of the activity is fitted to a quadratic function (solid line) with an apparent *K*_I_ = 30 ± 8 nm at a saturating concentration of 1.0 ± 0.1 μm PG.

The rate of dsDNA produced during steady‐state turnover of KF exo^−^ was initially investigated using a continuous assay with a saturating concentration (1 mm) of dNTPs and 100 nm of the T7pR30 template with a two‐fold excess of T7 primer annealed. With annealed T7 primer, the T7pR30 template has a 5′ overhang of 30 nucleotides that can be filled in during the reaction. The assay was performed in the presence of PG and the change in [dsDNA] was quantified by monitoring the fluorescence emission from PG at 525 nm. PG was found to strongly inhibit the reaction with an apparent *K*_I_ = 30 ± 8 nm (Fig. 3). It is notable that this value is of the same order as the *K*_d_ reported for DNA binding to PG 34 and of that determined in Fig. 2. Similar inhibition has been reported previously 14 and can be rationalized if polymerase binding to the template DNA is inhibited when template DNA has bound PG present; essentially, PG appears to be acting like a competitive inhibitor. While it may be possible to perform continuous assays in the presence of low concentrations (sub‐μm) of PG, this is problematic. In order for PG to linearly report on [dsDNA], it must be saturating relative to the final amount of dsDNA produced. With 100 nm of a 50‐mer template (as in Fig. 3), a minimum PG concentration of 1.25 μm is required to allow PG to bind, on average, to every fourth base pair of dsDNA 35. If a longer template is used (see later), then more PG is required and/or the concentration of template must be reduced, which reduces the sensitivity of the assay. This is also assuming that intercalation is the largest contributor to fluorescence enhancement/emission, given that PG can also associate with DNA via electrostatics and groove binding 35.

**Figure 4:**
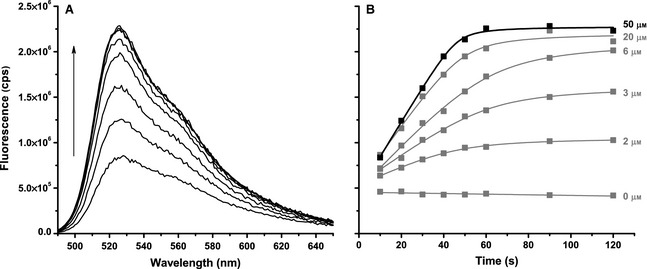
Stopped steady‐state assay. (A) Fluorescence emission spectra obtained during a stopped steady‐state assay of KF exo^−^ with T7pR30. The spectra correspond to the 50 μm data in (B) shown in black. (B) Reaction progress curves measured using the fluorescence maxima at each time point and dNTP concentration (as labelled). All measurements were performed with 50 nm KF exo^−^ and 100 nm 1 : 1.2 T7pR30 : T7 primer (annealed) at room temperature.

As an alternative to optimizing the conditions of the continuous assay, a stopped assay was explored. The reaction conditions were similar to those used in Fig. 3 and typically contained 10 or 100 nm primed template and 0–500 μm dNTPs. The reaction was initiated by the addition of 5 or 50 nm KF exo^−^ and aliquots were quenched by the addition of 3 μm PG at 10 s intervals. We used PG to quench the reaction as EDTA (a common polymerase quencher) was found to reduce the fluorescence of PG/DNA solutions. This is most probably due to EDTA sequestering metal ions (i.e. Mg^2+^), which have been shown to enhance PG fluorescence 45. No activity was measured with 3 μm PG in Fig. 3, and care was taken during the stopped assay to minimize the time between quenching and fluorescence measurement. The stopped assay with T7pR30 template is shown in Fig. 4. The reaction progress curves show the reaction going to completion with higher dNTP concentrations. In these cases, the template is limiting and the reaction stops when the single‐strand 5′ overhang of the template is filled in. This was confirmed by performing assays with progressively longer templates with 50, 80 and 100 nucleotides of 5′ overhang (Table 1; data not shown). These data showed the reaction saturating after increasingly longer intervals, with correspondingly longer linear regions and higher final fluorescence levels. One additional advantage of a stopped assay is that the concentration of the reaction can be diluted upon quenching in order to ensure that PG is saturating. Thus, it is possible to use longer templates without compromising the sensitivity of the assay.

As the progress curves measured are non‐linear, these data were fitted to a quadratic function to extract the initial velocities (Eqn (5)) with example fits shown in Fig. 4B.

(5) $$\Delta f=\left(V_{\mathrm{initial}}/2\right)\left\{\left(c+t_{\mathrm{sat}}+t\right)-\sqrt{{\left(c+t_{\mathrm{sat}}+t\right)}^{2}-4t_{\mathrm{sat}}t}\right\}$$

Equation (5) provides a model‐free method of fitting non‐linear time course data in order to determine the slope of the initial linear region, i.e. *V*_initial_. The *t*_sat_ and *c* parameters are phenomenological and describe the time when the reaction has saturated (~ 60 s in Fig. 4B) and the curvature around this point, respectively.

**Figure 5:**
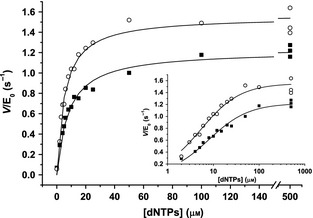
Michaelis–Menten steady‐state data. The dNTP dependence of the observed initial reaction velocity data extracted from stopped assays of KF exo^−^ (50 nm) with 100 nm T7pR50 (circles) and T7pR100 (squares) templates. The inset shows the same data on a log concentration axis and the data are fitted to Eqn (6) with fitted values given in Table 2.

The initial velocity data show a hyperbolic dependence of the observed velocity on dNTP concentration (Fig. 5). By fitting these data to a Michaelis–Menten expression (Eqn (6)), apparent *V*_max_ and *K*_m_ values were determined for each of the four templates (Fig. 5; Table 2).

(6) $$V_{\mathrm{obs}}=V_{\max}\left[\mathrm{dNTP}\right]/\left(K_{m}+\left[\mathrm{dNTP}\right]\right)$$

**Table 2:** Apparent Michaelis–Menten parameters derived from fits of Eqn (6) (with fitting errors) to stopped steady‐state assays of the dNTP dependence of KF exo^−^ with 50 nm template.

Template	*k*_cat_ (s^−1^)	*K*_m_ (μm)	*k*_cat_/*K*_m_ (μm^−1^·s^−1^)
T7pR30	1.43 ± 0.05	4.2 ± 0.6	0.34 ± 0.06
T7pR50	1.55 ± 0.03	5.3 ± 0.5	0.29 ± 0.03
T7pR80	0.93 ± 0.03	2.8 ± 0.5	0.33 ± 0.07
T7pR100	1.22 ± 0.02	8.0 ± 0.6	0.15 ± 0.02

As template was in excess over enzyme in these experiments, apparent *k*_cat_ values were determined by dividing *V*_max_ by the concentration of polymerase. While there is some variability in the measured *k*_cat_ and *K*_m_ values, they are similar to literature values reported using steady‐state methods 4. Interestingly, *k*_cat_/*K*_m_, which is a measure of productive dNTP capture, is significantly less variable. The variability in *k*_cat_ and *K*_m_ values may arise due to sequence variations between our templates. In our experiments, 30–100 nucleotides of dsDNA were synthesized (depending on the template) when the assay was allowed to go to completion.

**Figure 6:**
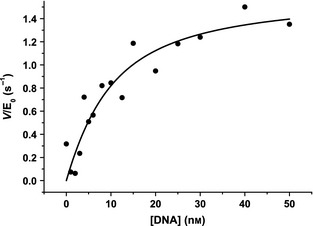
Pseudo‐Michaelis–Menten steady‐state data. The T7pR80 template DNA dependence of the observed initial reaction velocity data extracted from stopped assays of 5 nm KF exo^−^ with 500 μm dNTPs. The data are fitted to Eqn (7) (*E*_0_ = 5 nm) to obtain *V*_max_ = 1.6 ± 0.2 s^−1^ and *K*_m_ = 7 ± 3 nm.

The dependence of the T7pR80 template concentration on the stopped assay was next explored by performing the assay with saturating dNTPs (500 μm). KF exo^−^ was used at 5 nm to allow sufficient time to acquire a reasonable reaction time course (data not shown). These data were again fitted to Eqn (5) to extract initial velocities that were then fitted to a ‘tight‐binding’ expression (Eqn (7); [E]_0_ is the initial enzyme concentration) to extract apparent *V*_max_ and *K*_m_ values for DNA binding to the polymerase (Fig. 6).

(7) $$V=\left(V_{\max}/2{\left[E\right]}_{0}\right)\left\{\left({\left[E\right]}_{0}+{\left[\mathrm{dsDNA}\right]}_{0}+K_{m}\right)-\sqrt{{\left({\left[E\right]}_{0}+{\left[\mathrm{dsDNA}\right]}_{0}+K_{m}\right)}^{2}-4{\left[E\right]}_{0}{\left[\mathrm{dsDNA}\right]}_{0}}\right\}$$

Values of *V*_max_ = 1.6 ± 0.2 s^−1^ and *K*_m_ = 7 ± 3 nm were determined from the data in Fig. 6.

**Figure 7:**
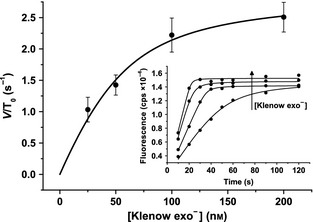
Pseudo‐Michaelis–Menten steady‐state data. The enzyme (KF exo^−^) dependence of the observed initial reaction velocity data extracted from stopped assays with 50 nm T7pR100 template and 500 μm dNTPs. The data are fitted to Eqn (7) (*T*_0_ = 50 nm) to obtain *V*_max_ = 2.9 ± 0.3 s^−1^ and *K*_m_ = 22 ± 10 nm. The inset shows the time course of the experiments performed with 25, 50, 100 and 200 nm KF exo^−^. These data are fitted to Eqn (5) in order to extract the *V*_obs_ values plotted in the main panel.

Finally, the dependence of the KF exo^−^ concentration on the stopped assay was investigated with 50 nm T7pR100 template and saturating dNTPs (500 μm). It became progressively more difficult to extract reliable initial velocities from the time courses as the enzyme concentration was increased, as the reaction reached completion more quickly (Fig. 7, inset). The enzyme concentration dependence on the observed velocity appears to show saturation behaviour (Fig. 7) and these data were fitted to Eqn (7) to determine apparent *V*_max_ = 2.9 ± 0.3 s^−1^ and *K*_m_ = 22 ± 10 nm values. It is interesting to note that the apparent *K*_m_ values determined from Figs 6 and 7, respectively, are not significantly different and are similar to values determined for the association of KF with DNA using pre‐steady‐state methods 3. It may be possible to study the processivity of the DNA polymerase using this assay, but a rapid‐quench approach is likely to be required if significantly shorter templates are to be used.

In order for an enzyme reaction to be at steady‐state, it has been argued that the following condition, ε, must be met: ε = *E*_0_/(*K*_m_ + *S*_0_) ≪ 1 48. While this criterion may not be strictly valid for non‐Michaelis–Menten mechanisms such as that of DNA polymerase (which is multi‐step and effectively has two substrates), it can still aid in the interpretation of enzyme turnover data. The dNTP dependences (Fig. 4; Table 2) were measured under conditions where ε < 0.01, while the template and enzyme dependences were, by necessity, measured with 0.08 < ε < 0.7 and 0.3 < ε < 3.2, respectively. Consequently, it would appear that our assay is capable of measuring steady‐state turnover of DNA polymerase but only for nucleotide incorporation. This is likely to be a problem for other steady‐state assays, and it is interesting to note that the value of *k*_cat_ determined in Fig. 6 is similar to those determined from the dNTP dependences (Table 2).

In this report, we have demonstrated a quantitative steady‐state kinetic assay of the stoichiometric conversion of ssDNA to dsDNA catalysed by DNA polymerases, based on the differential fluorescence of PG when bound to dsDNA and ssDNA. PG was found to be a potent inhibitor of the KF exo^−^ reaction with an apparent inhibition constant of ~ 30 nm. However, by performing a stopped assay, Michaelis–Menten parameters (*k*_cat_, *K*_m_, *k*_cat_/*K*_m_) for DNA polymerase KF exo^−^ were determined and found to be in good agreement with previously reported values. The assay was found to be reliable over a range of DNA, enzyme and nucleotide concentrations and offers a viable alternative to conventional kinetic assays of DNA polymerase that employ radionucleotides. We also see no reason why this stopped assay cannot be applied to other DNA polymerase enzymes (and possibly reverse transcriptase enzymes), although the inhibition of the enzyme by PG should be confirmed first or an alternative quencher used. By employing a fluorescence plate reader, it is anticipated that a full steady‐state assay of an enzyme could be performed in a few minutes. This could then lead to a screening assay, not only to examine enzyme kinetics, but potentially also to look at, for example, polymerase inhibitors.

## Materials and methods

KF exo^−^ and NEB buffer 2 (10 mm Tris/HCl, 50 mm NaCl, 10 mm MgCl_2_, 1 mm dithiothreitol, pH 7.9) were obtained from New England Biolabs (Hitchin, Hertfordshire, UK). KF exo^−^ was monitored between batches by SDS/PAGE (single band at ~ 68 kDa) and its concentration was determined spectrophotometrically (ε_280_ = 55 450 m^−1^·cm^−1^) 43. dNTPs were obtained from Peqlab Ltd (Sarisbury Green, Hampshire, UK). PG was obtained from Invitrogen (Life Technologies, Paisley, UK) and its concentration was determined by ε_500_ = 70 000 m^−1^·cm^−1^ 36. PG was always used at a concentration below 4 μm for fluorescence experiments to avoid inner filter effects.

HPLC‐purified DNA oligonucleotides were obtained from Eurofins MWG Operon (London, UK) and their sequences are given in Table 1. The sequences are designed to anneal to the T7 primer (from the T7 promoter sequence, explaining their nomenclature), leaving a 30–100 nucleotide 5′ overhang. This region of ssDNA is randomized and has approximately equal ratios of A : T : C : G. No secondary structure is predicted in any of the sequences. Oligonucleotides were annealed by heating the mixture to 95 °C for 10–15 min in buffer 2 (see below) before cooling to room temperature over at least 45 min. Unless stated otherwise, primed templates used for steady‐state kinetics were made using a template oligo : T7 primer ratio of 1 : 1.2.

Fluorescence measurements were performed with an Edinburgh Instruments FLS920 spectrometer (Edinburgh, UK) with a 450 W xenon arc lamp light source, double excitation and emission monochromators and a red‐sensitive cooled photomultiplier detector. Quartz fluorescence cells (Starna Scientific Ltd, Hainault, UK) with a 100 μL cell volume and 10 mm excitation pathlength were used. All measurements were performed at room temperature (~ 23 °C). Care was taken to minimize photobleaching of PG by minimizing the excitation energy using a 0.5 nm excitation slit width. For all measurements, a 5 nm emission slit width and 0.1 s averaging time were used when acquiring spectra.

Steady‐state reactions were performed in NEB buffer 2. The standard assay consisted of 100 nm DNA template, 5 or 50 nm polymerase and equal quantities of each dNTP (combined concentrations are given in the text). Reactions were typically initiated by the addition of the polymerase. Discontinuous assays were stopped with 3 μm PG and measurements were made within 10 min of stopping. No significant change in fluorescence was observed upon waiting longer before measurement.

## References

[1] Lehman IR, Bessman MJ, Simms ES & Kornberg A (1958) Enzymatic synthesis of deoxyribonucleic acid. I. Preparation of substrates and partial purification of an enzyme from *Escherichia coli*. J Biol Chem 233, 163–170 13563462

[2] Bryant FR, Johnson KA & Benkovic SJ (1983) Elementary steps in the DNA polymerase I reaction pathway. Biochemistry 22, 3537–3546 6351905 10.1021/bi00284a001

[3] Kuchta RD, Mizrahi V, Benkovic PA, Johnson KA & Benkovic SJ (1987) Kinetic mechanism of DNA polymerase I (Klenow). Biochemistry 26, 8410–8417 3327522 10.1021/bi00399a057

[4] Polesky AH, Steitz TA, Grindley ND & Joyce CM (1990) Identification of residues critical for the polymerase activity of the Klenow fragment of DNA polymerase I from *Escherichia coli*. J Biol Chem 265, 14579–14591 2201688

[5] Richardson CC, Schildkraut CL, Aposhian HV & Kornberg A (1964) Enzymatic synthesis of deoxyribonucleic acid. XIV. Further purification and properties of deoxyribonucleic acid polymerase of *Escherichia coli*. J Biol Chem 239, 222–232 14114848

[6] Bertram JG, Oertell K, Petruska J & Goodman MF (2010) DNA polymerase fidelity: comparing direct competition of right and wrong dNTP substrates with steady state and pre‐steady state kinetics. Biochemistry 49, 20–28 20000359 10.1021/bi901653g PMC2803633

[7] Johnson KA (1995) Rapid quench kinetic analysis of polymerases, adenosinetriphosphatases, and enzyme intermediates. Methods Enzymol 249, 38–61 7791620 10.1016/0076-6879(95)49030-2

[8] Mizrahi V, Benkovic P & Benkovic SJ (1986) Mechanism of DNA polymerase I: exonuclease/polymerase activity switch and DNA sequence dependence of pyrophosphorolysis and misincorporation reactions. Proc Natl Acad Sci USA 83, 5769–5773 3016719 10.1073/pnas.83.16.5769 PMC386376

[9] Mizrahi V, Benkovic PA & Benkovic SJ (1986) Mechanism of the idling‐turnover reaction of the large (Klenow) fragment of *Escherichia coli* DNA polymerase I. Proc Natl Acad Sci USA 83, 231–235 3510431 10.1073/pnas.83.2.231 PMC322831

[10] Mizrahi V & Benkovic SJ (1988) The dynamics of DNA polymerase‐catalyzed reactions. Adv Enzymol Relat Areas Mol Biol 61, 437–457 2833078 10.1002/9780470123072.ch8

[11] Mizrahi V, Henrie RN, Marlier JF, Johnson KA & Benkovic SJ (1985) Rate‐limiting steps in the DNA polymerase I reaction pathway. Biochemistry 24, 4010–4018 3902078 10.1021/bi00336a031

[12] Joyce CM (2010) Techniques used to study the DNA polymerase reaction pathway. Biochim Biophys Acta 1804, 1032–1040 19665596 10.1016/j.bbapap.2009.07.021 PMC2846202

[13] Schwartz JJ & Quake SR (2009) Single molecule measurement of the ‘speed limit’ of DNA polymerase. Proc Natl Acad Sci USA 106, 20294–20299 19906998 10.1073/pnas.0907404106 PMC2787106

[14] Seville M, West AB, Cull MG & McHenry CS (1996) Fluorometric assay for DNA polymerases and reverse transcriptase. Biotechniques 21, 664, 666, 668, 670, 672. 8891218 10.2144/96214st04

[15] Tveit H & Kristensen T (2001) Fluorescence‐based DNA polymerase assay. Anal Biochem 289, 96–98 11161300 10.1006/abio.2000.4903

[16] Ma CB, Tang ZW, Wang KM, Tan WH, Li J, Li W, Li ZH, Yang XH, Li HM & Liu LF (2006) Real‐time monitoring of DNA polymerase activity using molecular beacon. Anal Biochem 353, 141–143 16624242 10.1016/j.ab.2006.02.006

[17] Yu LM, Hu GL & Howells L (2002) Fluorescence‐based, high‐throughput DNA polymerase assay. Biotechniques 33, 938–941 12398204 10.2144/02334pf02

[18] Griep MA (1995) Fluorescence recovery assay: a continuous assay for processive DNA polymerases applied specifically to DNA polymerase III holoenzyme. Anal Biochem 232, 180–189 8747473 10.1006/abio.1995.0005

[19] LePecq JB & Paoletti C (1967) A fluorescent complex between ethidium bromide and nucleic acids. Physical‐chemical characterization. J Mol Biol 27, 87–106 6033613 10.1016/0022-2836(67)90353-1

[20] Morgan AR, Lee JS, Pulleyblank DE, Murray NL & Evans DH (1979) Review: ethidium fluorescence assays. Part I. Physicochemical studies. Nucleic Acids Res 7, 547–569 41222 10.1093/nar/7.3.547 PMC328039

[21] Morgan AR, Evans DH, Lee JS & Pulleyblank DE (1979) Review: ethidium fluorescence assays. Part II. Enzymatic studies and DNA−protein interactions. Nucleic Acids Res 7, 571–594 388344 10.1093/nar/7.3.571 PMC328040

[22] Purohit V, Grindley ND & Joyce CM (2003) Use of 2‐aminopurine fluorescence to examine conformational changes during nucleotide incorporation by DNA polymerase I (Klenow fragment). Biochemistry 42, 10200–10211 12939148 10.1021/bi0341206

[23] Frey MW, Sowers LC, Millar DP & Benkovic SJ (1995) The nucleotide analog 2‐aminopurine as a spectroscopic probe of nucleotide incorporation by the Klenow fragment of *Escherichia coli* polymerase I and bacteriophage T4 DNA polymerase. Biochemistry 34, 9185–9192 7619819 10.1021/bi00028a031

[24] Holden MJ, Haynes RJ, Rabb SA, Satija N, Yang K & Blasic JR Jr (2009) Factors affecting quantification of total DNA by UV spectroscopy and PicoGreen fluorescence. J Agric Food Chem 57, 7221–7226 19627145 10.1021/jf901165h

[25] Koba M, Szostek A & Konopa J (2007) Limitation of usage of PicoGreen dye in quantitative assays of double‐stranded DNA in the presence of intercalating compounds. Acta Biochim Pol 54, 883–886 18066405

[26] Blotta I, Prestinaci F, Mirante S & Cantafora A (2005) Quantitative assay of total dsDNA with PicoGreen reagent and real‐time fluorescent detection. Ann Ist Super Sanita 41, 119–123 16037660

[27] Batchelor R, Hagen D, Johnson I & Beechem J (2003) A fluorescent high‐throughput assay for double‐stranded DNA: the RediPlate PicoGreen assay. Comb Chem High Throughput Screen 6, 287–291 12769671 10.2174/138620703106298536

[28] McGowan KB, Kurtis MS, Lottman LM, Watson D & Sah RL (2002) Biochemical quantification of DNA in human articular and septal cartilage using PicoGreen and Hoechst 33258. Osteoarthritis Cartilage 10, 580–587 12127839 10.1053/joca.2002.0794

[29] Zipper H, Brunner H, Bernhagen J & Vitzthum F (2004) Investigations on DNA intercalation and surface binding by SYBR Green I, its structure determination and methodological implications. Nucleic Acids Res 32, e103 15249599 10.1093/nar/gnh101 PMC484200

[30] Hadfield SJ & Chalmers RM (2012) Detection and characterization of *Cryptosporidium cuniculus* by real‐time PCR. Parasitol Res 111, 1385–1390 22392139 10.1007/s00436-012-2874-1

[31] Boehm D, Herold S, Kuechler A, Liehr T & Laccone F (2004) Rapid detection of subtelomeric deletion/duplication by novel real‐time quantitative PCR using SYBR‐green dye. Hum Mutat 23, 368–378 15024731 10.1002/humu.20011

[32] Abiodun OO, Gbotosho GO, Ajaiyeoba EO, Happi CT, Hofer S, Wittlin S, Sowunmi A, Brun R & Oduola AM (2010) Comparison of SYBR Green I‐, PicoGreen‐, and [3H]‐hypoxanthine‐based assays for *in vitro* antimalarial screening of plants from Nigerian ethnomedicine. Parasitol Res 106, 933–939 20165881 10.1007/s00436-010-1743-z

[33] Medina RA, Rojas M, Tuin A, Huff S, Ferres M, Martinez‐Valdebenito C, Godoy P, Garcia‐Sastre A, Fofanov Y & SantaLucia J Jr (2011) Development and characterization of a highly specific and sensitive SYBR green reverse transcriptase PCR assay for detection of the 2009 pandemic H1N1 influenza virus on the basis of sequence signatures. J Clin Microbiol 49, 335–344 21084522 10.1128/JCM.01142-10 PMC3020443

[34] Cosa G, Focsaneanu KS, McLean JR, McNamee JP & Scaiano JC (2001) Photophysical properties of fluorescent DNA‐dyes bound to single‐ and double‐stranded DNA in aqueous buffered solution. Photochem Photobiol 73, 585–599 11421063 10.1562/0031-8655(2001)073<0585:PPOFDD>2.0.CO;2

[35] Dragan AI, Casas‐Finet JR, Bishop ES, Strouse RJ, Schenerman MA & Geddes CD (2010) Characterization of PicoGreen interaction with dsDNA and the origin of its fluorescence enhancement upon binding. Biophys J 99, 3010–3019 21044599 10.1016/j.bpj.2010.09.012 PMC2965993

[36] Singer VL, Jones LJ, Yue ST & Haugland RP (1997) Characterization of PicoGreen reagent and development of a fluorescence‐based solution assay for double‐stranded DNA quantitation. Anal Biochem 249, 228–238 9212875 10.1006/abio.1997.2177

[37] Tolun G & Myers RS (2003) A real‐time DNase assay (ReDA) based on PicoGreen fluorescence. Nucleic Acids Res 31, e111 12954787 10.1093/nar/gng111 PMC203337

[38] Kuchta RD, Benkovic P & Benkovic SJ (1988) Kinetic mechanism whereby DNA polymerase I (Klenow) replicates DNA with high fidelity. Biochemistry 27, 6716–6725 3058205 10.1021/bi00418a012

[39] Kuchta RD, Cowart M, Allen D & Benkovic SJ (1988) Kinetic and structural investigations of the replicative fidelity of the Klenow fragment. Biochem Soc Trans 16, 947–949 3224760 10.1042/bst0160947

[40] Dahlberg ME & Benkovic SJ (1991) Kinetic mechanism of DNA polymerase I (Klenow fragment): identification of a second conformational change and evaluation of the internal equilibrium constant. Biochemistry 30, 4835–4843 1645180 10.1021/bi00234a002

[41] Eger BT & Benkovic SJ (1992) Minimal kinetic mechanism for misincorporation by DNA polymerase I (Klenow fragment). Biochemistry 31, 9227–9236 1327109 10.1021/bi00153a016

[42] Benkovic SJ & Cameron CE (1995) Kinetic analysis of nucleotide incorporation and misincorporation by Klenow fragment of *Escherichia coli* DNA polymerase I. Methods Enzymol 262, 257–269 8594352 10.1016/0076-6879(95)62022-2

[43] Joyce CM & Grindley ND (1983) Construction of a plasmid that overproduces the large proteolytic fragment (Klenow fragment) of DNA polymerase I of *Escherichia coli*. Proc Natl Acad Sci USA 80, 1830–1834 6340110 10.1073/pnas.80.7.1830 PMC393703

[44] Joyce CM, Kelley WS & Grindley ND (1982) Nucleotide sequence of the *Escherichia coli* polA gene and primary structure of DNA polymerase I. J Biol Chem 257, 1958–1964 6276402

[45] Dragan AI, Bishop ES, Casas‐Finet JR, Strouse RJ, Schenerman MA & Geddes CD (2010) Metal‐enhanced PicoGreen fluorescence: application to fast and ultra‐sensitive pg/ml DNA quantitation. J Immunol Methods 362, 95–100 20833180 10.1016/j.jim.2010.09.011

[46] Dragan AI, Bishop ES, Casas‐Finet JR, Strouse RJ, Schenerman MA & Geddes CD (2010) Metal‐enhanced PicoGreen fluorescence: application for double‐stranded DNA quantification. Anal Biochem 396, 8–12 19748479 10.1016/j.ab.2009.09.010

[47] Polesky AH, Dahlberg ME, Benkovic SJ, Grindley ND & Joyce CM (1992) Side chains involved in catalysis of the polymerase reaction of DNA polymerase I from *Escherichia coli*. J Biol Chem 267, 8417–8428 1569092

[48] Segel LA (1988) On the validity of the steady‐state assumption of enzyme‐kinetics. Bull Math Biol 50, 579–593 3219446 10.1007/BF02460092

